# Factors associated with magnetic resonance imaging defined patellar tendinopathy in community-based middle-aged women: a prospective cohort study

**DOI:** 10.1186/s12891-015-0645-8

**Published:** 2015-08-05

**Authors:** Jason Toppi, Jessica Fairley, Flavia M. Cicuttini, Jill Cook, Susan R. Davis, Robin J. Bell, Fahad Hanna, Yuanyuan Wang

**Affiliations:** Department of Epidemiology and Preventive Medicine, Monash University, Alfred Hospital, Melbourne, VIC 3004 Australia; Department of Physiotherapy, School of Primary Health Care, Faculty of Medicine, Nursing and Health Sciences, Monash University, Frankston, VIC 3199 Australia; Women’s Health Research Program, School of Public Health and Preventive Medicine, Monash University, Alfred Hospital, Melbourne, VIC 3004 Australia

**Keywords:** Patellar tendinopathy, Physical activity, Vastus medialis, Magnetic resonance imaging

## Abstract

**Background:**

Patellar tendinopathy identified by imaging modalities has been reported in asymptomatic athletes and associated with tendon-related symptoms. However there is little data in community-based populations. The aim of this cohort study was to examine the prevalence of magnetic resonance imaging (MRI) defined patellar tendinopathy, the factors associated with this condition, and whether it was associated with knee pain in community-based middle-aged women.

**Methods:**

One hundred seventy six women, aged 40–67 years, with no significant knee pain or injury underwent knee MRI. Patellar tendinopathy was defined on both T1- and T2-weighted fat-saturated MRIs. The cross-sectional area of vastus medialis was measured from MRI. Height and weight were measured to calculate body mass index (BMI). Physical activity was assessed using a questionnaire. Knee pain was assessed using the Western Ontario and McMaster University Osteoarthritis Index.

**Results:**

The prevalence of MRI defined patellar tendinopathy was 30.1 %. Higher levels of physical activity (odds ratio 1.65, 95 % CI 1.09–2.51) and greater vastus medialis cross-sectional area (odds ratio 1.22, 95 % CI 1.04–1.43) were associated with increased prevalence of patellar tendinopathy, independent of age and BMI. The persistence of patellar tendinopathy was associated with the worsening of knee pain over 2 years (odds ratio 10.65, 95 % CI 1.14–99.77).

**Conclusion:**

In community-based middle-aged women MRI-diagnosed patellar tendinopathy is common, with higher levels of physical activity and greater vastus medialis size being risk factors suggesting a biomechanical effect. Persistent patellar tendinopathy is associated with worsening of knee pain. These findings suggest that further work is needed to determine the contribution of patellar tendinopathy on knee pain and function in older people.

## Background

Optimizing knee function in older people is important. It is increasingly clear that causes of knee pain are multifactorial. Despite the evidence for a significant contribution of patellofemoral diseases to knee pain and disability [1], a significant focus of work has been on the tibiofemoral compartment with little work at the patellofemoral compartment. Patellofemoral pathology tends to cause knee pain on performing functional activities involving knee bending which are not well captured in most commonly used instruments which examine knee pain.

Patellar tendinopathy is a clinical condition characterised by activity-related anterior knee pain [2, 3]. It is commonly known as ‘jumper’s knee’ as it is traditionally recognised in athletes who are involved in sports that require jumping [3, 4]. However, patellar tendinopathy has also been found in people who do not participate in sports involving jumping [3–6]. The prevalence of patellar tendinopathy varied among athletes participating in different sports, with values ranging between 2.5 and 45 % [6–8]. The diagnosis of patellar tendinopathy is primarily based on clinical examination. However patellar tendon abnormalities identified by imaging modalities such as magnetic resonance imaging (MRI) and ultrasonography have been shown to be corresponded to the characterised pathological features of patellar tendinopathy histopathologically [5, 9, 10]. Although not all individuals with evidence of patellar tendinopathy on imaging have symptoms [5, 11], there is evidence that abnormalities identified by imaging modality within the asymptomatic tendon predict the development of tendon-related symptoms and disability [12, 13].

Currently there is little data on the prevalence and factors associated with patellar tendinopathy in community-based non-athletic populations, and it is unknown whether patellar tendinopathy contributes to knee symptoms in older people. Understanding these is important as patellar tendinopathy may contribute to activity-related knee pain which may impair the functional activities of the knee [14]. We have recently reported the prevalence (28.3 %) of MRI defined patellar tendinopathy in community-based older individuals with past and current obesity being the major risk factor [15]. However the relationship between patellar tendinopathy and knee pain is not fully investigated [15]. Given the mechanical mechanism in the pathogenesis the patellar tendinopathy [3], physical activity [16] and quadriceps strength [2] have been recognized as risk factors for patellar tendinopathy in athletes, although the evidence is not strong [17]. It is not known whether physical activity and quadriceps strength are risk factor for patellar tendinopathy in community-based older populations. The cross-sectional area of a muscle has been validated as a measure of the force producing capability of that muscle [18, 19] and could be used as a surrogate for muscle strength. Thus the aim of this study was to (1) determine whether MRI defined patellar tendinopathy was prevalent, (2) identify factors associated with this condition, and (3) examine whether patellar tendinopathy was associated with knee pain, in a cohort of community-based middle-aged women. We hypothesised that (1) MRI defined patellar tendinopathy will be common, (2) higher levels of physical activity and greater quadriceps muscle size will be associated with increased prevalence of patellar tendinopathy, and (3) patellar tendinopathy will be associated with knee pain in community-based women.

## Methods

### Study participants

Study participants of the current prospective cohort study were recruited from a previous cross-sectional study of 1423 women examining the role of androgens in sexual function using a database established from the electoral roll in Victoria, Australia, between April 2002 and August 2003 [20]. Women from the original cross-sectional study were eligible for the current prospective cohort study if they were aged 40 to 67 years, had not undergone a hysterectomy and agreed to be re-contacted for further studies. Of the 355 women who fulfilled these criteria, 176 remained eligible after further exclusion for significant knee pain lasting more than 24 h in the past 5 years that necessitated treatment by a doctor or physiotherapist, knee injury in the last 5 years requiring non-weight-bearing treatment for more than 24 h or surgery, any arthritis diagnosed by a medical practitioner, any contraindication to MRI scan including pacemaker, metal sutures, presence of shrapnel or iron filings in the eye, or claustrophobia, or being unlikely to be available to complete the longitudinal protocol of re-assessment at 2 years [21]. The study was approved by the Southern Health Human Research and Ethics Committee and the Monash University Human Research and Ethics Committee. All participants gave written informed consent.

### Anthropometric, physical activity, and clinical measurements

Weight was measured to the nearest 0.1 kg using electronic scales with shoes, socks, and bulky clothing removed. Height was measured to the nearest 0.1 cm using a stadiometer with shoes and socks removed. Body mass index (BMI) (weight/height^2^, kg/m^2^) was then calculated from these measurements. Physical activity was assessed using a questionnaire in 3 categories: walking (1 = <0.5 miles/week; 2 = 0.5–5 miles/week; 3 = 5–10 miles/week; 4 = >10 miles/week), work (1 = sedentary; 2 = sedentary and occasional exercise; 3 = 50 % sedentary and 50 % active; or active housework; 4 = predominantly manual active all day), and sport (1 = none; 2 = 1 h/week of golf, bowls, badminton, cycling, or swim; 3 = >2 h/week of above activities; or >1 h/week of fitness, aerobics, or squash; 4 = >2 h/week of fitness, aerobics, or squash). A total physical activity score was created by adding up the above scores, ranging from 1 to 12 [22], and were further categorized into tertiles. Participants with scores of 1–6 were deemed to have ‘low levels of physical activity’, those with scores of 7–8 deemed to have “moderate levels of physical activity”, and those with scores of 9–12 deemed to have ‘high levels of physical activity’. Knee pain was assessed at the time of knee MRI (baseline and approximately 2 years later), by Western Ontario and McMaster University Osteoarthritis Index (WOMAC) [23]. The pain subscale comprises five questions, each of which is assessed on a 100 mm visual analogue scale and summed to give a total pain score out of 500. Increase in the score corresponds with worsening of pain. The incidence of knee pain was defined as baseline WOMAC knee pain score of 0 and knee pain score of more than 50 at 2 years later. The worsening of knee pain was defined as any increase in WOMAC knee pain score of more than 20 over 2 years.

### MRI

An MRI of each participant’s dominant knee, defined as the leg from which the participant stepped off from when initiating gait, was performed between October 2003 and August 2004 [21] and approximately 2 years later. Knees were imaged in the sagittal plane on a 1.5-T whole body magnetic resonance unit (Philips Medical Systems, Eindhoven, The Netherlands) using a commercial transmit-receive extremity coil. The following sequence and parameters were used: a T1-weighted fat suppressed 3D gradient recall acquisition in the steady state, flip angle 55 °, repetition time 58 msec, echo time 12 msec, field of view 16 cm, 60 partitions, 512 × 512 matrix, one acquisition time 11 min 56 sec. Sagittal images were obtained at a partition thickness of 1.5 mm and an in-plane resolution of 0.31 × 0.31 mm (512 × 512 pixels). In addition, a coronal T2-weighted fat-saturated acquisition, repetition time 2200 msec, echo time 20/80 msec, slice thickness 3.0 mm, 1.0 mm interslice gap, one excitation, field of view 11 to 12 cm and matrix of 256 × 128 pixels was obtained.

### Assessment of patellar tendinopathy

**Fig. 1:**
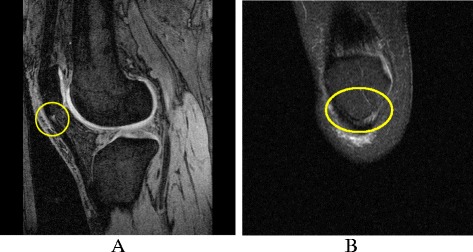
Patellar tendinopathy on MRI. **a** Sagittal T1-weighted fat-saturated image showing patellar tendinopathy. **b** Coronal T2-weighted fat-saturated image showing patellar tendinopathy

Patellar tendinopathy was defined as an area of increased signal intensity of characteristic pattern, size and distribution on at least two adjacent slices in the proximal region of the inferior patellar tendon. Two trained observers, who were blinded to participant characteristics, assessed the presence of patellar tendinopathy for each participant on T1-weighted fat-saturated sagittal images. T2-weighted fat-saturated coronal images were used to confirm the presence of patellar tendinopathy and ensure a magic angle effect was not contributing to positive results. When echo time is short, increased signal intensity can be observed in the presence of no pathology. This artificially increased intensity can potentially be confused with disease and is known as the magic angle effect. To overcome the magic angle effect, a T2-weighted sequence can be applied [24], which was done in this study. The patellar tendon was graded as either ‘definite tendinopathy’ or ‘no tendinopathy’ [11, 24, 25] (Fig. 1). The intra-observer and inter-observer reproducibility for determination of definite patellar tendinopathy, assessed using 50 randomly selected knee MRI scans, was 0.94 and 0.90 (expressed as intra-class correlation coefficient, ICC), respectively. The persistence of patellar tendinopathy was defined as those who had ‘definite tendinopathy’ at both baseline and 2 years later.

### Measurement of vastus medialis cross-sectional area

Vastus medialis cross-sectional area was measured from axial MR images reformatted from sagittal images, as previously described [26]. The distal vastus medialis cross-sectional area was measured directly from these axial images by a trained observer by manually drawing disarticulation contours around the muscle boundaries using the independent workstation software Osiris (Digital Imaging Unit, University Hospital of Geneva, Geneva, Switzerland). The cross-sectional area of vastus medialis was measured at the MR slice 37.5 mm superior to the quadriceps tendon insertion at the proximal pole of the patella. This slice was orthogonal to the long axis of the leg. One trained observer measured the vastus medialis cross-sectional area in duplicate, 1 week apart, blinded to the participant characteristics. The average of the two measures was taken as the final results. The intra-observer reliability for the vastus medialis cross-sectional area measurement (expressed as ICC) was 0.99.

### Statistical analysis

Descriptive statistics for the characteristics of the study participants were tabulated. Continuous variables were compared using independent samples *t*-test or Mann–Whitney *U* test where appropriate, and categorical variables were compared using chi squared test. Logistic regression was used to examine the association between exposures of interest (independent variables–vastus medialis cross-sectional area and physical activity) and the prevalence of MRI defined patellar tendinopathy at baseline (dependent variable), adjusted for potential confounders of age and BMI. Logistic regression was used to examine the association between the prevalence/persistence of MRI defined patellar tendinopathy (independent variable) and the change on knee pain over 2 years (dependent variable), adjusted for age and BMI. *P*-values of less than 0.05 were considered statistically significant. All analyses were performed using the IBM SPSS statistics 20.

## Results

The characteristics of the 176 women are summarized in Table 1. The women were largely asymptomatic, which was evidenced by the very low score of the WOMAC pain subscale at the time of knee MRI, with the median scores being 21 out of 500. Fifty-three (30.1 %) women had MRI defined patellar tendinopathy. There were no significant differences in terms of age, BMI, vastus medialis cross-sectional area, physical activity, and WOMAC pain score between those with and without patellar tendinopathy.

**Table 1:** Characteristics of study participants

	No tendinopathy (*N* = 123)	Definite tendinopathy (*N* = 53)	*P* value*
Age^a^, years	52.2 (7.1)	52.5 (5.6)	0.80
Body mass index^a^, kg/m^2^	27.1 (5.4)	27.1 (5.7)	0.94
Vastus medialis cross-sectional area^a^, mm^2^	1047 (180)	1130 (293)	0.06
Physical activity score^b^, *n* (%)			0.07
Low (1–6)	53 (44.2)	16 (30.8)	
Moderate (7–8)	39 (32.5)	15 (28.8)	
High (9–12)	28 (23.3)	21 (40.4)	
WOMAC pain score^c^	21 (0–240)	21 (0–311)	0.92

Physical activity data was available for 172 women

*For difference between those with and without patellar tendinopathy using independent samples t test^a^, chi squared test^b^, or Mann–Whitney U test^c^

Data presented as mean (SD)^a^, number (%)^b^, or median (range)^c^

In univariate analysis, vastus medialis cross-sectional area and physical activity levels were positively associated with the prevalence of MRI defined patellar tendinopathy. After adjustment for age and BMI, greater vastus medialis cross-sectional area (OR per cm^2^ = 1.22, 95 % CI 1.04–1.43, *p* = 0.02) and higher levels of physical activity (OR per tertile = 1.65, 95 % CI 1.09–2.51, *p* = 0.02) remained to be associated with increased prevalence of patellar tendinopathy (Table 2). When vastus medialis cross-sectional area and physical activity were included in the same regression model and adjusted for age and BMI, the association for vastus medialis cross-sectional area remained significant (OR 1.19, 95 % CI 1.01–1.40, *p* = 0.04) but the association for physical activity was attenuated and no longer significant (OR 1.52, 95 % CI 0.99–2.32, *p* = 0.06).

**Table 2:** Factors associated with the prevalence of MRI defined patellar tendinopathy

	Univariate analysis	*P* value	Multivariate analysis	*P* value
	Odds ratio (95 % CI)		Odds ratio (95 % CI)	
Age (years)^a^	1.01 (0.96, 1.06)	0.82	1.01 (0.96, 1.06)	0.82
Body mass index (kg/m^2^)^b^	1.00 (0.95, 1.06)	0.94	1.00 (0.94, 1.06)	0.95
Cross-sectional area of vastus medialis (cm^2^) ^c^	1.18 (1.02, 1.37)	0.03	1.22 (1.04, 1.43)	0.02
Physical activity (tertiles)^c^	1.58 (1.05, 2.36)	0.03	1.65 (1.09, 2.51)	0.02

^a^Adjusted for body mass index; ^b^adjusted for age; ^c^adjusted for age and body mass index

A total of 148 women had knee MRI at 2 year follow-up, of whom 46 women had patellar tendinopathy at baseline. Over 2 years, the persistence of patellar tendinopathy was observed in 20 out of 46 (43.5 %) participants. The incidence of knee pain was 3 out of 46 (6.5 %), and the worsening knee pain was 22 out of 148 (14.9 %). The association between MRI defined patellar tendinopathy and changes in knee pain over 2 years was examined (Table 3). Although there was no significant association between the prevalence of patellar tendinopathy and the incidence of knee pain (OR 6.46, 95 % CI 0.49–85.68, *p* = 0.16), the persistence of patellar tendinopathy was associated with the worsening of knee pain (OR 10.65, 95 % CI 1.14–99.77, *p* = 0.04).

**Table 3:** Association between MRI defined patellar tendinopathy and change in knee pain over 2 years

	Univariate analysis	*P* value	Multivariate analysis^c^	*P* value
	Odds ratio (95 % CI)		Odds ratio (95 % CI)	
Prevalence of patellar tendinopathy	5.82 (0.48, 70.62)^a^	0.17	6.46 (0.49, 85.68)^a^	0.16
Persistence of patellar tendinopathy	10.71 (1.17, 98.24)^b^	0.04	10.65 (1.14, 99.77)^b^	0.04

^a^Incidence of knee pain (baseline pain score = 0 & follow-up pain score >50) over 2 years

^b^Worsening of knee pain (any increase in WOMAC pain score of >20) over 2 years

^c^Adjusted for age and BMI

## Discussion

MRI defined patellar tendinopathy was common in community-based middle-aged women without clinical knee diseases, with a prevalence of 30.1 %. Both higher levels of physical activity and greater vastus medialis size were associated with greater prevalence of MRI defined patellar tendinopathy. The findings suggest that being physically active may be a risk factor for patellar tendinopathy, indicating a mechanical pathogenesis of patellar tendinopathy. Furthermore, persistent patellar tendinopathy predicted the worsening of knee pain over 2 years.

Imaging, in particular ultrasonography defined patellar tendinopathy has been described in asymptomatic young athletes [12, 13]. It has been shown to be common and predict the development of knee symptoms and future tendon-related disability [12, 13]. There is little literature characterising patellar tendinopathy in community-based populations. Recently we reported a prevalence of 28.3 % for MRI defined patellar tendinopathy in community-based older adults with obesity being the risk factor [15]. In the current study of community-based in middle-aged women, we found that the prevalence of MRI defined patellar tendinopathy was 30.1 %. These findings suggest that imaging identified patellar tendinopathy is a common condition occurring in the general populations but not just a condition of athletes [2, 6, 17]. Our study found an increased risk of patellar tendinopathy in relation to higher levels of physical activity and greater vastus medialis size. Previously it has been shown in athletes that the number of jumps performed correlated with an increased risk of patellar tendinopathy [16] and that there was an association between quadriceps muscle strength and patellar tendinopathy [17]. Our results in a community-based population support these findings. Moreover, we showed that the positive association between vastus medialis size and patellar tendinopathy was independent of the level of physical activity. In contrast to our previous study showing obesity being a risk factor for MRI defined patellar tendinopathy in community-based older adults [15], we found no relationship between patellar tendinopathy and BMI in middle-aged women in the current study. Evidence is not consistent across the literature. In athletes an association has been identified between patellar tendinopathy and increased body weight, waist circumference, and BMI [2, 6]. However, some argue that weight is not a risk factor for the development of patellar tendinopathy [27, 28], which is in keeping with the findings of this study. Taken together, these results suggest that MRI defined patellar tendinopathy is prevalent in community-based women and is associated with both muscle size (a surrogate of quadriceps muscle strength) and levels of physical activities. Although in this study there are no measures of intermediate variables which are more representative of knee biomechanics, our findings indicate a mechanical rather than systemic metabolic pathogenesis of patellar tendinopathy. This is consistent with the conclusion from a systematic review of the risk factors for patellar tendinopathy that all nine identified risk factors affect the loading of the patellar tendon in some way, and could be explained within the framework of a mechanical pathophysiological theory [17].

Although the clinical significance of ultrasonography identified patellar tendinopathy has been reported in asymptomatic young athletes [12, 13], it is unknown for the clinical implications of the MRI defined patellar tendinopathy in community-based populations. We found that persistent patellar tendinopathy was associated with the worsening of knee pain. However the wide confidence interval for the significant association can be explained by the moderate sample size of the study. Our study did not show a significant association between the prevalence of patellar tendinopathy and the incidence of knee pain (*p* = 0.16). Our study sample had a low incidence of knee pain (6.5 %) which limited the power of our study to show a significant result. Larger prospective cohort studies with longer follow-up will be needed to further explore the relationship between MRI defined patellar tendinopathy and knee symptoms.

There are several limitations in our study. The sample size of our study is modest. Further research of larger prospective cohort studies should be conducted to assess the natural history of patellar tendinopathy over time and its clinical significance in relation to knee symptoms. Although we collected WOMAC data at two time points, we did not specifically ask about knee pain on bending, which would be more specific for patellar tendinopathy. This study investigated the risk factors for MRI diagnosed patellar tendinopathy in community-based women without significant knee pain. Thus the findings may not be generalizable to men or people with clinical patellar tendinopathy. A strength of our study is that patellar tendinopathy has been measured using the non-invasive MRI technique based on both T1- and T2-weighted sequences which showed high reproducibility, and analysis and disease classification have been conducted using well validated methodologies.

Patellofemoral compartment pathology has been recognised as a source of pain and impaired function with ageing [1]. It has been found that knee pain and functional limitations in activities such as knee bending, which have significant impact on knee function and quality of life, are common in older people. Understanding the modifiable risk factors for knee pain in community-based older populations will have the potential to inform preventive and therapeutic strategies for reducing knee pain and improving knee function. Symptomatic patellar tendinopathy has negative impacts on activity and quality of life [14]. Most of the instruments currently used in epidemiological studies of older people focus on the tibiofemoral compartment and do not address symptoms that may be attributable to patellar tendinopathy, such as activity-related anterior knee pain associated with important functional activities such as knee bending. Our study suggests that patellar tendinopathy is common in community-based middle-aged women. Further work using specific tools will be needed to determine whether patellar tendinopathy contributes to some of the common functional impairment seen in older people but not captured by the commonly used knee instruments.

## Conclusions

Our study has shown that MRI defined patellar tendinopathy is common in community-based middle-aged women. Patellar tendinopathy is associated with mechanical factors including physical activity and vastus medialis muscle size. The persistence of MRI defined patellar tendinopathy predicts the worsening of knee pain over 2 years. These findings suggest a mechanical pathogenesis and the clinical significance of MRI defined patellar tendinopathy. Further work in larger cohort studies will be needed to confirm the contribution of MRI defined patellar tendinopathy on knee joint pain and function in community-based populations.
